# Left ventricular resynchronization with left bundle branch area pacing: does the type of capture matter?

**DOI:** 10.1093/europace/euad152

**Published:** 2023-06-09

**Authors:** Karol Curila, Pugazhendhi Vijayaraman

**Affiliations:** Cardiocenter, Third Faculty of Medicine and Faculty Hospital Kralovske Vinohrady, Prague, Czechia; Geisinger Heart Institute, Geisinger Commonwealth School of Medicine, 1000 E Mountain Blvd, MC 36-10, Wilkes Barre, PA 18711, USA

## Abstract

**Graphical Abstract:**
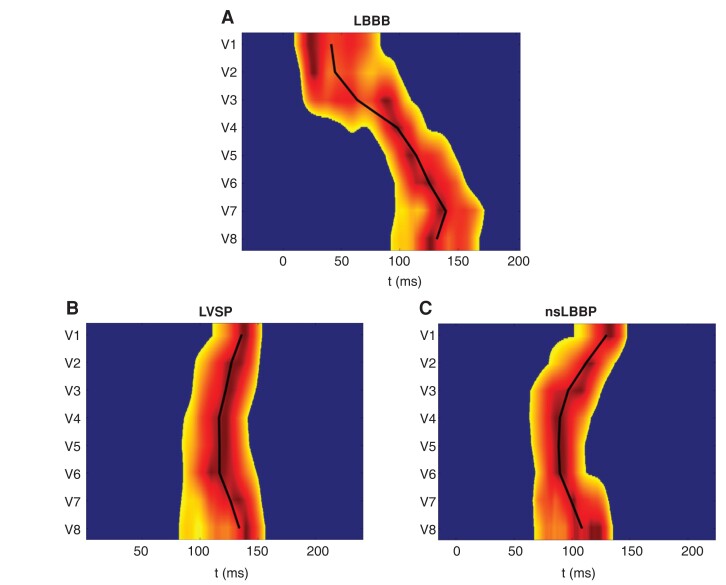
Visualization of ventricular activation sequence by using an ultra-high-frequency ECG in heart failure patient with LBBB and LV ejection fraction of 32% (A). LBBB was corrected by LVSP (*B*), and nsLBBP (*C*). Both LVSP and nsLBBP corrected right-to-left ventricular dyssynchrony present during LBBB (e-DYS 93 ms). They led to left-to-right ventricular activation pattern, with e-DYS—20 ms during LVSP and—41 during nsLBBP. e-DYS, time difference between the first and last UHF-ECG activation; for more details, see Jurak *et al*.^12^ LVSP, left septal myocardial pacing.

Visualization of ventricular activation sequence by using an ultra-high-frequency ECG in heart failure patient with LBBB and LV ejection fraction of 32% (A). LBBB was corrected by LVSP (*B*), and nsLBBP (*C*). Both LVSP and nsLBBP corrected right-to-left ventricular dyssynchrony present during LBBB (e-DYS 93 ms). They led to left-to-right ventricular activation pattern, with e-DYS—20 ms during LVSP and—41 during nsLBBP. e-DYS, time difference between the first and last UHF-ECG activation; for more details, see Jurak *et al*.^12^ LVSP, left septal myocardial pacing.

**This editorial refers to ‘Stepwise application of ECG and electrogram-based criteria to ensure electrical resynchronization with left bundle branch pacing’, by M. Pujol-López *et al*., https://doi.org/10.1093/europace/euad128.**

Cardiac resynchronization therapy (CRT) with biventricular pacing (BiV) has been a mainstream treatment in heart failure patients with wide QRS complexes for two decades.^1^ In recent years, left bundle branch pacing (LBBP) and left septal myocardial pacing (LVSP) appeared as two novel CRT strategies. During LBBP, there is direct left bundle branch (LBB) capture, while during LVSP, myocytes of the left septum are captured first, and the left ventricular (LV) conduction system is activated with some latency. The common term left bundle branch area pacing (LBBAP) is used to reflect the deep septal deployment of the pacing lead with direct or indirect capture of the LBB fibres.^2^

Currently, only limited clinical evidence comparing these new pacing techniques to biventricular CRT exists. Early data show that in patients with non-ischaemic cardiomyopathy and left bundle branch block (LBBB), LBBP is superior to BiV CRT with respect to the level of electromechanical resynchronization, acute haemodynamic response,^3^ and LV ejection fraction improvement after 6 months.^4^ Left septal myocardial pacing, performed by an electrophysiology (EP) catheter by a retrograde transaortic approach, provided better electrical resynchronization and a similar increase in d*T*/d*t*_max_ as BiV CRT.^5^

Although these results have shown a promising effect of LBBAP, there are several pitfalls associated with their straightforward adoption as the mainstream treatment in CRT patients. First, we do not have enough data on their effect in ischaemic cardiomyopathy patients and those with more distal LV conduction defects, i.e. catheter Intraventricular conduction delay (IVCD). More importantly, we do not have the criteria allowing the implanting physician to clearly understand the capture type during the implant procedure in all CRT patients. Criteria for LVSP performed by transseptal approach were never introduced, and those for LBBP presented by Huang *et al*.^6^ might not be demonstrable in all CRT patients. The late r/R in V1 is not specific for LBB capture, as it just reflects earlier LV than RV activation. Moreover, it might be missing during LBB capture in patients with peripheral LV conduction defects, which prolong and postpone LV activation. The transition from non-selective to selective LBB capture (nsLBBp to sLBBP) or LVSP during the decremental output pacing might remain unrecognized or even not present in patients with equal capture thresholds for LBB and septal myocardium. The duration of paced V_6_ R-wave peak time (RWPT) is not specific for any capture type in CRT patients. It may be significantly shorter during the LVSP in patients with proximal LBBB and healthy peripheral Purkinje conduction than during LBB capture in patients with severely dilated LV and/or diseased distal LV conduction system.

Left bundle branch area pacing is not an easy procedure, even for skilled implanters.^2,7^ The success rate of LBBAP was reported to be 92% in bradycardia patients but only 82% in patients with heart failure and a need for CRT.^7^ Moreover, the dominant type of capture was fascicular pacing, with LBB trunk capture in only 9% of patients and LVSP in ∼25% of included patients.^8^ Recognizing the variability in LBB anatomy, the diversity of aetiologies of heart failure, and various degrees of disease progression is crucial for understanding a long learning curve and the difficulties in recognizing the capture type during LBBAP in CRT patients. Therefore, it is reasonable to ask if we should strictly care about the type of ventricular capture or focus on the primary objective, which is the electrical resynchronization of dyssynchronous ventricular activation.

In the context of these facts, Pujol-López *et al*.^9^ present interesting data which shed more light on the effect of LBBAP on reducing ventricular dyssynchrony in CRT-eligible patients. They have studied LV resynchronization by measuring the LV activation time (LVAT) using electrocardiography imaging (ECGi) in a cohort of 24 patients in which LBBAP was attempted in the LEVEL-AT trial.^9^ Most patients had wide QRS complex, predominantly LBBB, and on average, the LV ejection fraction was 27%. In addition, one-third of patients had ischaemic cardiomyopathy. They showed that LV resynchronization was achieved in 22 patients, in which the LVAT was reduced on average for 40 ± 17 ms, with a minimum shortening of 17 ms during LBBAP.

Interestingly, only in 13 patients, a qR or rSR pattern was present in V1. In eight other patients, LV resynchronization was achieved while paced QRS complex had a V1 QS pattern, and concomitantly QRS duration (measured from the first to last deflection) was shorter ≤120 ms (LBBC-Plus criterion). In the last patient with LV resynchronization, neither qR/rSR in V1 nor LBBC-Plus was present, but paced V_6_RWPT was <80 ms. There was a lack of electrical resynchronization after LBBAP in two patients, in whom compared with patients with successful LV resynchronization, LVAT post-pacing was longer by 32 ms. Finally, when they analysed which criteria would predict LV electrical resynchronization, they found the combination of a late qR/rSR in V1 and LBBC-Plus criterion outperformed others, with accuracy reaching 96%.

This study adds more insight into the CRT treatment in patients with heart failure. It shows that even without the commonly used criteria for LBB capture^6^ or LVSP (deep septal position of the pacing lead and qR/rSR in V1), pacing in the left septal area results in LV resynchronization compared with a spontaneous LBBB or RV pacing. This observation is, however, not surprising as both LBBP and LVSP eliminate a transseptal conduction delay which is the main determinant of postponed LV activation in patients with proximal LBBB or RV septal pacing^10–12^ (*Graphical Abstract*). In bradycardia patients, both LBBP and LVSP result in left-to-right activation pattern, with faster LV activation during LBBP but more balanced ventricular depolarization during LVSP.^11,13,14^ We still miss such data in heart failure patients, and the recent work of Pujol-López *et al*. does not provide them. But it shows that in CRT-eligible patients, the LV dyssynchrony may be improved even without the left-to-right activation pattern on 12-lead ECG or the presence of other criteria for LBB capture. These are very important findings regarding the results of the primary analysis of the study by Pujol-López *et al*.^9^ It showed that LBBAP led to a greater electrical resynchronization and similar echocardiographic response compared with BiV CRT. It would be interesting to know if the patients without qR/rSR had the same, better, or worse LV resynchronization than those with qR/rSR in V1 and if the level of the dyssynchrony reduction during LBBAP correlated with the degree of the LV ejection fraction improvement. Finally, knowing the relation of the pacing leads’ tip to the LV endocardium in patients with QS patterns in V1, would allow us to better understand if the more balanced ventricular activation during LBBAP was the result of diseased LV conduction or if the lead tips were placed in the shallower deep septal positions. They may produce synchronous ventricular activation with a shorter QRS duration than during the left septal or left bundle branch pacing.^15^

## Data Availability

All relevant data are within the manuscript and its supporting file.
